# T-Follicular Helper Cells and Their Role in Autoimmune Diseases

**DOI:** 10.3390/life15040666

**Published:** 2025-04-17

**Authors:** Michalis Christodoulou, Eleni Moysidou, Georgios Lioulios, Stamatia Stai, Christina Lazarou, Aliki Xochelli, Asimina Fylaktou, Maria Stangou

**Affiliations:** 1 School of Medicine, Aristotle University of Thessaloniki, Department of Nephrology, Hippokration Hospital, Konstantinoupoleos 49, 54642 Thessaloniki, Greece; michalischristodoulou22@gmail.com (M.C.); moysidoueleni@yahoo.com (E.M.); pter43@yahoo.gr (G.L.); staimatina@yahoo.gr (S.S.); lachristine91@yahoo.com (C.L.); 2 Department of Immunology, National Histocompatibility Center, Hippokration General Hospital, 54642 Thessaloniki, Greece; aliki.xochelli@gmail.com (A.X.); fylaktoumina@gmail.com (A.F.)

**Keywords:** autoimmune diseases, T-follicular helper cells, cytokines, vasculitis, systemic lupus erythematosus, rheumatoid arthritis

## Abstract

T-follicular helper (Tfh) cells, a specialized subset of CD4^+^ cells, are the immune mediators connecting cellular and humoral immunity, as they lead B-cell proliferation within germinal centers, and orchestrate their response, including activation, class switching, and production of a diverse array of high-affinity antibodies. Their interactions with B cells is regulated by a wide complex of transcriptional and cytokine-driven pathways. A major contribution of Tfh cells to autoimmune diseases is through their production of cytokines, particularly IL-21, which supports the proliferation and differentiation of autoreactive B cells. Elevated levels of circulating Tfh-like cells and IL-21 have been observed in patients with systemic lupus erythematosus (SLE) and rheumatoid arthritis (RA) correlating strongly with disease severity and autoantibody levels. The feedback loop between Tfh cells and IL-21 or other signal pathways, such as Bcl-6, ICOS, and PD-1, not only sustains Tfh cell function but also drives the continuous expansion of autoreactive B cells, leading to chronic inflammation through the production of high-affinity pathogenic autoantibodies. By understanding these interactions, Tfh pathways may serve as potential therapeutic targets, with IL-21, ICOS, and PD1 blockades emerging as promising innovative therapeutic strategies to manage autoimmune diseases. Although a variety of studies have been conducted investigating the role of Tfh cells in SLE and RA, this review aims to reveal the gap in the literature regarding the role of such subpopulations in the pathogenesis of other autoimmune diseases, such as Anca-associated vasculitis (AAV), and express the need to conduct similar studies. Tfh cell-related biomarkers can be used to assess disease activity and transform autoimmune disease treatment, leading to more personalized and effective care for patients with chronic autoimmune conditions.

## 1. Introduction

T-follicular helper (Tfh) cells stand as a specialized subset of CD4^+^ lymphocytes and their main role is to initiate and regulate B-cell responses [1]. They serve as the connecting link between cellular and humoral immunity. Based on their ability to interact and activate B lymphocytes, Tfh cells have emerged as critical regulators of humoral immunity, supporting B-cell differentiation and antibody production [2]. These interactions between Tfh and B lymphocytes take place within the germinal centers (GC) of secondary lymphoid organs, such as spleen and lymph nodes [3].

Therefore, Tfh cells are essential for the formation and maintenance of GC, where they assist B cells undergo processes such as affinity maturation and class switching to produce high-affinity antibodies [4].

However, while Tfh cells are vital for protective immune responses, their dysregulation can lead to the production of autoreactive antibodies, driving the pathogenesis of autoimmune diseases. In this review, we will analyze their functions in normal conditions and explore critical changes in their behavior in autoimmune diseases, with a focus on SLE, RA, and AAV, their contribution to, and the emerging insights into ANCA-associated vasculitis (AAV). We will also discuss potential therapeutic strategies aimed at targeting Tfh cells or their key signaling pathways [1,4,5].

One of the central players in this finely tuned process is the Tfh cells, which represent a specialized subset of CD4^+^ T cells that closely interact with B cells to promote the production of high-affinity antibodies [4,5]. Tfh cells are essential for the formation and maintenance of GC, the specialized microenvironment sites within lymphoid organs, where B lymphocytes undergo a process called somatic hypermutation, and class switching, ensuring the production of a diverse array of antibodies that can effectively neutralize pathogens in certain types of immune responses [6].

However, Tfh cells are not only essential to initiating B-lymphocyte activation but also to effectively maintaining immune homeostasis by guiding B-cell responses in the production of high-affinity, class-switched antibodies against pathogens only, and prevent them from excessive autoimmune responses [7].

Importantly, recent studies have expanded our understanding of B-cell activation by highlighting alternative, Tfh-independent mechanisms. Certain T-cell subsets—such as Th1 and Th17 cells—can provide help to B cells outside of the germinal center microenvironment, contributing to autoantibody production in a GC-independent manner [7,8]. These extrafollicular responses may be particularly relevant in chronic inflammatory conditions where dysregulated immune activation bypasses traditional checkpoints.

Nevertheless, in the context of autoimmune diseases, this delicate balance in autoimmune reactions, and the competition between defensive and destructive responses is often disrupted, almost always leading to excessive B-cell activation. Tfh cells seem to play a central role in these mechanisms of unbalanced reactions [8]. In addition, the role of Age-related B cells (ABCs)—a distinct B-cell subset capable of forming spontaneous, aberrant germinal center-like structures—has gained increasing attention. ABCs can reciprocally induce dysregulated Tfh responses, further amplifying autoimmune cascades through persistent T–B-cell interactions [9]. Deregulated Tfh-cell activity can lead to the production of autoantibodies, chronic inflammation, and tissue damage. There are several findings that highlight the implication and involvement of Tfh cells in the pathogenesis of certain autoimmune diseases, including systemic lupus erythematosus (SLE), rheumatoid arthritis (RA), and anti-neutrophil cytoplasmic antibody-associated vasculitis (AAV), not only by upregulating the production of harmful autoantibodies, but also by preserving the inflammatory and autoimmune cascades [9]. Understanding the mechanisms by which Tfh cells contribute to the pathophysiology of these diseases is crucial in their abrupt intervention and long-term management. By investigating their complicated and multifaceted mechanisms, researchers intend to identify new therapeutic approaches that aim to regulate and possibly restrain Tfh cell function, without compromising the body’s ability to mount normal immune responses.

In addition to their roles in autoimmune diseases, Tfh cells have been implicated in the pathogenesis of angioimmunoblastic T-cell lymphoma (AITL), a rare but increasingly recognized lymphoma. AITL arises from aberrant Tfh-cell activity, where these cells acquire malignant properties and promote lymphoid tissue proliferation and immune dysregulation. The rising incidence of AITL underscores the importance of understanding Tfh-cell biology not only in autoimmune settings but also in cancer immunology [10].

The present review describes the function of Tfh cells, their important role in maintaining adaptive immune system activity, and their detrimental role in certain autoimmune systemic diseases. Although a variety of studies have been conducted investigating the role of Tfh cells in SLE and RA, this review aims to emphasize the gap in the existing literature regarding the role of such subpopulations in the pathogenesis of AAV.

## 2. Phenotypic Characteristics of Tfh Cells

The main characteristic that distinguishes Tfh cells from other CD4^+^ subpopulations is their unique transcriptional program and their ability to interact with B cells in the context of GC. The differentiation between Naïve CD4 lymphocytes and Tfh cells is driven by the transcription factor Bcl-6, which represses the gene programs of other T helper (Th) cell subsets and promotes the expression of key molecules, including CXCR5, ICOS**, and PD-1, essential to defining the Tfh phenotype [11].

Tfh cells exhibit phenotypic diversity, often classified into Tfh1, Tfh2, and Tfh17 subtypes, each associated with specific cytokine profiles and immune responses [1,10,12,13].

Tfh1 cells are characterized by the production of IFN-γ; they are involved in pro-inflammatory responses and are implicated in supporting IgG1 and IgG3 isotype switching in B cells. This subtype is often upregulated in autoimmune diseases, such as RA, where IFN-γ-driven inflammation exacerbates joint damage [10,12,13].

Tfh2 cells produce IL-4 and are linked to IgE and IgG4 class switching, playing a role in allergic responses and some autoimmune diseases. IL-4 from Tfh2 cells supports humoral responses while limiting excessive inflammation, making them potentially protective in certain contexts [14].

Tfh17 cells are defined by IL-17A production and are associated with inflammatory autoimmune diseases such as multiple sclerosis (MS) and RA. IL-17A enhances neutrophil recruitment, furthering tissue damage in autoimmune settings. Studies suggest that Tfh17 cells may contribute to AAV pathogenesis, where IL-17-driven inflammation underlines vascular injury [12,13].

Importantly, despite their cytokine diversity, all Tfh subsets—Tfh1, Tfh2, and Tfh17—share the ability to produce interleukin-21 (IL-21), a hallmark cytokine critical for B-cell help, germinal center formation, and antibody class switching. While IL-21 is also produced by Th17 cells, particularly in peripheral inflammatory sites, its high and sustained expression is a defining feature of Tfh cells, distinguishing them from other helper T-cell subsets [14]. This shared IL-21 production reinforces the central role of Tfh cells in humoral immunity, irrespective of their specialized functional phenotype.

The introduction of Tfh-cell subtypes, including Tfh1, Tfh2, and Tfh17, has provided a framework to understand the nuanced roles of these cells in health and diseases. Each phenotype produces unique cytokine profiles that contribute to different immune pathways, with implications for autoimmunity and targeted treatment. For instance, Tfh1 cells, which produce IFN-γ, are often associated with heightened inflammation in RA, while Tfh17 cells, which produce IL-17, contribute to tissue inflammation in multiple sclerosis and potentially AAV. These phenotypes reflect the adaptability of Tfh cells in response to environmental signals, offering pathways to develop phenotype-specific therapies for autoimmune diseases [14,15]. By selectively modulating Tfh1, Tfh2, or Tfh17 cells, it may be possible to target specific aspects of immune dysregulation without compromising broader immune functions.

### 2.1. Key Markers and Signaling Pathways

**Figure 1:**
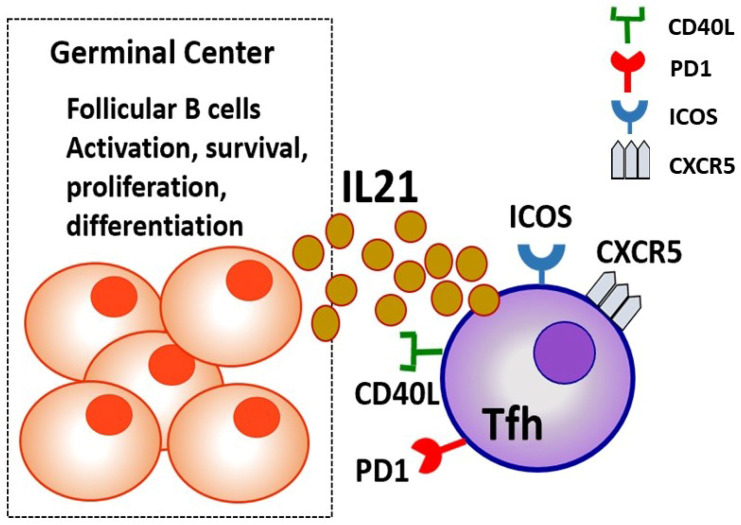
T-follicular helper (Tfh) cell migration and function within germinal centers (GCs) of lymphoid organs. Naïve CD4^+^ T cells are primed by dendritic cells in the T-cell zone and differentiate into pre-Tfh cells. These cells upregulate CXCR5 and migrate toward the B-cell follicle. Within germinal centers, Tfh cells interact with B cells to support their selection, proliferation, and affinity maturation. Tfh cells express key markers such as PD-1, Bcl6, and ICOS, and secrete IL-21. A subset of Tfh cells exits the GCs to become circulating Tfh cells, which may contribute to systemic humoral responses.

The expression of certain surface molecules that characterize Tfh cells are as follows: CXC chemokine receptor type 5 (CXCR5) is a chemokine receptor expressed on Tfh cells that allows their migration to B-cell follicle regions and GCs of secondary lymphoid organs. CXCR5 molecule is the main receptor that determines Tfh certain role in B-cell activation, through binding to CXCL13 molecule, a chemokine highly expressed on B follicular cells [16]. Inducible T-cell Costimulator (ICOS) is crucial for the initiation of Tfh-cell differentiation and their continued activation of B cells. ICOS acts as a co-stimulatory molecule as it supports interaction between T and B lymphocytes, promotes cytokine production, and enhances the survival and proliferation of both cell types [17]. Programmed Death-1 (PD-1) serves as a regulatory molecule, controlling the magnitude of the Tfh-cell response by preventing overactivation and maintaining immune homeostasis. Elevated PD-1 expression is a marker of fully differentiated Tfh cells within GCs. CD40 ligand (CD40L) on their surface, which binds to CD40 on B lymphocytes, an interaction, which apart from B-cell activation, leads to B-cell differentiation into memory B cells and plasma cells [18]. The Signaling Lymphocyte Activation Molecule (SLAM-1, CD150) is a receptor that helps interactions between Tfh and B cells. Tfh cells are characterized by the downregulation of the CCR7 surface molecule expression. Low CCR7 expression allows Tfh cells to travel from the T-cell zones into the B-cell follicles of lymphoid organs where they can exert their function [19], as shown in Figure 1.

#### Structure of the B-Cell Follicle and Germinal Center

The B-cell follicle is a specialized region within secondary lymphoid organs (such as lymph nodes and the spleen) where B cells reside and respond to antigens. Within these follicles, a GC can form during an immune response [19]. GC is crucial for antibody affinity maturation and the differentiation of B cells into long-lived plasma cells and memory B cells. The GC consists of two main compartments: the Dark Zone, containing rapidly proliferating B cells known as centroblasts, specific cells undergoing somatic hypermutation (SHM) in their immunoglobulin (Ig) genes to improve antigen-binding affinity and the Light Zone which contains smaller, less proliferative B cells called centrocytes. These cells express mutated B-cell receptors (BCRs) and interact with follicular dendritic cells (FDCs) that present antigens. T-follicular helper (Tfh) cells provide crucial survival and differentiation signals via CD40L and cytokines like IL-21. B cells with high-affinity BCRs are positively selected for further differentiation [20].

## 3. Cytokine Production

Tfh cells produce a range of cytokines that are critical for their function within germinal centers, particularly in supporting B-cell activation. Among these, IL-21 plays a central role by promoting B-cell proliferation, survival, and differentiation into plasma cells and memory B cells. Beyond its effects on B cells, IL-21 also enhances the survival and expansion of Tfh cells themselves, establishing a positive feedback loop that sustains germinal center reactions and antibody production [21]. IL-4, produced by Tfh cells, supports class switching in B cells. In mice, it promotes switching to IgE and IgG1, while in humans, it primarily induces switching to IgE and IgG4. IL-10 production is less prominent in cytokine produced by Tfh cells and exerts a rather anti-inflammatory role [22].

## 4. Regulation of Tfh Cells

Tfh-cell differentiation and function are tightly regulated by a combination of intrinsic transcriptional programs and extrinsic signaling pathways from the microenvironment. Bcl-6 is the master regulator of Tfh-cell fate. It acts through the upregulation of CXCR5, ICOS, and PD-1, and the repression of transcription factors that promote Th1, Th2, and Th17 differentiation [23]. Several transcription factors, including Achaete-scute homolog 2 (Ascl2), signal transducers and activators of transcription (STAT1-5), c-Maf, BLIMP-1, Activating Transcription Factor 3 (ATF-3), Basic Leucine Zipper Transcription Factor (Batf), Interferon Regulatory Factors (IRF4 and IRF8), T-box expressed in T cells (T-bet), T cell-specific transcription factor 1 (TCF-1), and Lymphoid Enhancer Binding Factor 1 (LEF-1), play critical roles in Tfh-cell development and differentiation. In contrast, other factors, such as Bach2 (BTB and CNC Homology 2), Forkhead Box Protein O1 (FOXO1), Forkhead Box Protein P1 (FOXP1), and Krüppel-Like Factor 2 (KLF2), inhibit Tfh-cell differentiation by downregulating Bcl6 expression and reducing IL-21 production [22,23,24].

The interplay between these factors ensures that Tfh cells differentiate appropriately and function within the constraints of the immune response. Ascl2 is the transcription factor which is mainly responsible for the Tfh cells into B lymphocyte follicles, as they act in the early stages of Tfh-cell differentiation, by the upregulation of CXCR5 and repression of CCR receptor expression. Furthermore, reduced Blimp-1 expression, a molecule that antagonizes Bcl-6, prevents Tfh-cell inactivation [25].

Regulatory mechanisms, including PD-1 and T-follicular regulatory (Tfr) cells, help to limit the activity of Tfh cells, preventing excessive or autoreactive responses [26]. Tfr cells are a subset of regulatory T cells (Tregs) that express both CXCR5 and Bcl-6, allowing them to migrate to the GC and suppress Tfh-B cell interactions [27]. The dysregulation of these pathways can lead to unchecked Tfh-cell activity, contributing to autoantibody production and autoimmunity.

## 5. Differences Between Tfh Cells in GCs and Tfh Cells in Peripheral Circulation

**Figure 2:**
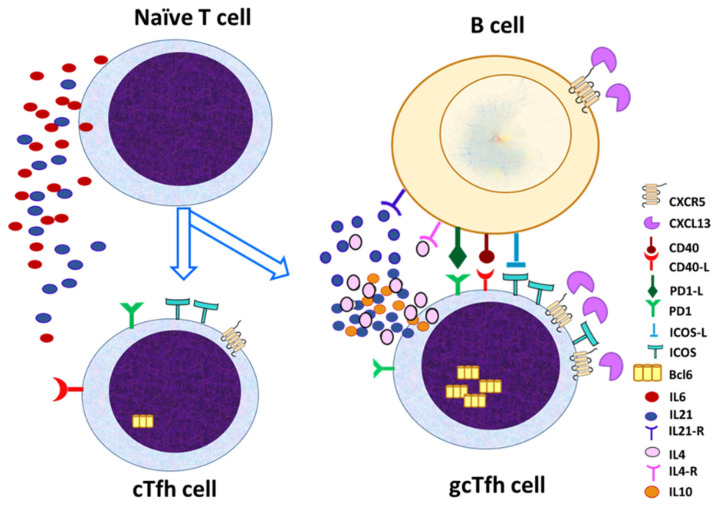
Differentiation and functional characteristics of Tfh-cell subsets. gcTfh cells, located in germinal centers (GCs), are characterized by high expression of CXCR5 and PD-1, and are influenced by cytokines like IL-6 and IL-21, along with transcription factors such as Bcl-6. These cells play a critical role in supporting B-cell maturation and affinity maturation. cTfh cells, present in peripheral circulation, share some phenotypic features with gcTfh cells but are less specialized and exhibit a more migratory and helper-like function. The distinction between these two populations is highlighted by differences in surface marker expression and functional roles in immune responses.

Tfh cells are primarily located in the B-cell follicles and germinal centers (GCs) of secondary lymphoid organs, referred to as gcTfh cells. These cells are driven by cytokines such as IL-6 and IL-21, as well as transcription factors, like Bcl-6, which support their differentiation into the germinal center environment. In contrast, Tfh cells are also found in peripheral circulation as cTfh cells [28,29]. These cTfh cells, while sharing some characteristics with their germinal center counterparts, are influenced by different signals and tend to have a more migratory and helper-like function. Although the two populations share similarities, they exhibit distinct phenotypic and functional characteristics, such as the expression of surface markers, like CXCR5 and PD-1, which are more pronounced in gcTfh cells (Figure 2).

The two populations exhibit distinct phenotypic and functional characteristics as shown in Table 1. Phenotypic differences include increased expression of CXCR5, PD-1, Bcl-6, and ICOS in the Tfh within the GC, while the peripheral Tfh cell may co-express other markers, such as CD45RO, which indicates their increased memory activity [28,29].

In steady-state conditions, circulating Tfh (cTfh) cells typically represent a small fraction of peripheral blood CD4^+^ T cells. Some researchers observed that the frequency of CD4^+^CXCR5+ICOS^high Tfh cells increased significantly in the peripheral blood of rheumatoid arthritis patients compared with healthy controls. Additionally, the absolute number of cTfh cells varies depending on individual and environmental factors, but it is generally low under baseline, non-inflammatory conditions. In lymph nodes, Tfh cells also represent a small percentage of total T cells, typically ranging between 5 and 10% in normal subjects. In cases of nodal benign hyperplasia, Tfh cells may be mildly expanded, but the percentage remains lower compared to pathological conditions, such as autoimmune diseases or malignancy. Understanding the baseline levels of Tfh cells in both peripheral blood and lymphoid tissues is essential for interpreting their dysregulation in autoimmune diseases or lymphoproliferative disorders [30].

## 6. Tfh Cells in Autoimmune Diseases

Tfh cells are instrumental in regulating B-cell responses within GCs of secondary lymphoid organs. They support B-cell maturation, somatic hypermutation, and isotype switching, which are processes essential for generating high-affinity antibodies. However, when dysregulated, Tfh cells can drive the production of autoantibodies, fueling the progression of autoimmune diseases. In systemic lupus erythematosus (SLE), rheumatoid arthritis (RA), and ANCA-associated vasculitis (AAV), aberrant Tfh-cell activity contributes to immune dysregulation and disease pathology [31,32,33].

### 6.1. Tfh Cells in Systemic Lupus Erythematosus (SLE) Pathogenesis

SLE is a complex autoimmune disease that affects multiple organs and is characterized by a wide range of autoantibodies, antinuclear, anti-dsDNA, antiRNP, and anti-Smith antibodies. The production of these autoantibodies in SLE patients is the direct consequence of the disrupted balance in Tfh-B-cell interactions within GCs and in circulation [31].

The levels of Tfh cells are increased in SLE patients, both within GCs and circulation, and play a central role in SLE pathogenesis. The dysregulation of Tfh cells has been closely associated with the development and progression of SLE. Several studies have reported an increased frequency of Tfh cells in the peripheral blood and lymphoid tissues of SLE patients, correlating with disease activity and autoantibody production. Recent studies have demonstrated that SLE patients exhibit a higher frequency of circulating Tfh (cTfh) cells compared to healthy controls. This elevation is often associated with increased disease activity, as measured by the SLE Disease Activity Index (SLEDAI). For instance, a study [34] reported that the frequency of cTfh cells was significantly elevated in active SLE patients and positively correlated with serum anti-double-stranded DNA (anti-dsDNA) antibody levels and SLEDAI scores. In addition to quantitative changes, qualitative alterations in Tfh cells have been observed in SLE. Specifically, an increased proportion of Tfh cells expressing inducible costimulator (ICOS) and programmed cell death protein 1 (PD-1) has been reported, indicating an activated phenotype. These activated Tfh cells are more efficient in providing B-cell help, potentially leading to enhanced autoantibody production [34,35].

Tfh cells from SLE patients often exhibit altered cytokine profiles, particularly the increased production of IL-21. IL-21 is a potent stimulator of B-cell differentiation into plasma cells and promotes class-switch recombination, processes that are dysregulated in SLE. Elevated serum IL-21 levels have been observed in SLE patients and are associated with higher disease activity and autoantibody titters. Other studies also demonstrated that blocking IL-21 signaling in a murine model of SLE resulted in reduced autoantibody production and amelioration of disease symptoms, highlighting the pathogenic role of IL-21 in SLE [36].

Several molecular pathways have been implicated in the aberrant expansion and function of Tfh cells in SLE. In SLE, the dysregulation of Bcl-6 expression has been observed, contributing to the abnormal expansion of Tfh cells. Moreover, other transcription factors, such as T-bet and STAT3, have been implicated in modulating Tfh-cell responses in SLE. For example, increased STAT3 activation has been reported in Tfh cells from SLE patients, promoting their differentiation and function [37]. Other studies also showed that inhibiting STAT3 signaling reduced Tfh-cell numbers and autoantibody production in an SLE mouse model [38].

Epigenetic changes, including DNA methylation and histone modifications, have been linked to Tfh-cell dysregulation in SLE. The hypomethylation of the IL21 gene promoter has been observed in Tfh cells from SLE patients, leading to increased IL-21 production [39,40,41,42]. Additionally, altered histone acetylation patterns have been reported in genes associated with Tfh cell function, suggesting that epigenetic therapies could be potential strategies for modulating Tfh-cell responses in SLE [39,40,41,42].

MicroRNAs (miRNAs) are small non-coding RNAs that regulate gene expression post-transcriptionally. The dysregulation of specific miRNAs has been implicated in Tfh-cell abnormalities in SLE. For instance, the reduced expression of miR-146a, a negative regulator of Tfh-cell differentiation, has been observed in SLE patients, leading to increased Tfh cell numbers and activity. Restoring miR-146a levels in a lupus mouse model resulted in decreased Tfh-cell responses and the amelioration of disease symptoms [40].

The interaction between Tfh cells and B cells is central to the pathogenesis of SLE. Aberrant Tfh–B-cell interactions contribute to the breakdown of B-cell tolerance and the production of pathogenic autoantibodies. In SLE, dysregulated Tfh-cell activity leads to the formation of hyperactive GCs, resulting in the generation of autoreactive B cells.

Although the role of both cTfh and gcTfh in the pathogenesis, progression, and sustained activity of SLE is indisputable, the distinct effects of cTfh cells subpopulations, cTfh1, cTfh2, and cTfh17, are still under investigation, and seem to be complicated, as various studies have demonstrated controversial results [40,41,42,43,44,45,46,47]. Novel SLE treatment strategies have just been released, aiming to focus on the deactivation and reduction in Tfh cells. Most in vitro and in vivo studies have focused on blocking the IL-21 cytokine. The first in vivo study targeting IL-21 in a lupus-prone MRL-Fas(lpr) mouse model—commonly used to study autoimmune diseases, such as lupus—was conducted more than 15 years ago, at a time when the production of IL-21 by Tfh cells had not yet been fully elucidated. Mice showed alleviation of skin lesions and lymphadenopathy, reduction in circulating dsDNA autoantibodies, IgG1 and IgG2a and urine protein levels, and improved histology after 10 weeks of treatment with IL-21R.Fc fusion protein. The same study also revealed that an IL21 blockade also reduced splenic T lymphocytes and reduced pathogenic autoantibodies production by splenic B lymphocytes [40,41,44,45,47].

Further studies in lupus-prone MRL/Mp-lpr/lpr (MRL/lpr) and B6.*Sle1.Yaa* mice revealed a beneficial effect after IL-21 neutralization in autoantibody production and also reduced renal-infiltrating Tfh and Th1 cells, improved renal histology, and reduced GC B cells and CD138^hi^ plasmablasts. These studies [40,41] demonstrated that Tfh cells and the derived IL-21 cytokine may orchestrate the autoreactive B lymphocyte production in SLE. However, a simultaneous reduction in IL-10 levels, a regulatory cytokine produced by Tfh cells under the influence of IL-21, significantly restricted the beneficial effects of IL-21. This finding revealed that Tfh cells, in addition to activating B lymphocytes, may also exert immune regulatory functions through IL-10 production [25,26]. Similarly, blocking ICOS activity with anti-B7RP-1 antibody (anti-B7RP-1 Ab) in a New Zealand Black/New Zealand White (NZB/NZW) F(1) mouse model of systemic lupus erythematosus led to a reduction in Tfh and gcB cells, which was accompanied by an improvement in disease manifestations [40,41].

Recently, the treatment of lupus-prone NZB/WF1 mice with CD40L antibodies or a combination CTLA4Ig and anti-CD40L reduced circulating B- and T lymphocytes and improved kidney inflammation [48]. Additionally, the use of circulating Tfh-cell levels as a biomarker may aid in monitoring disease activity and adjusting treatment regimens for patients with SLE. Recent studies have proven that peripheral T-follicular helper (Tfh) cells were increased during the early diagnosis of SLE, followed by a significant reduction during the first year of follow up [49]. Furthermore, Tf regulatory cells, which inhibit B-cell activation by Tfh cells, were reduced in patients with SLE, compared to healthy controls, and their levels showed a significant negative correlation with disease activity and serum anti-DNA levels. More interestingly though, both Tfr levels and the Tfr/Tfh ratio were improved to almost normal after adequate treatment [50].

### 6.2. Tfh Cells in Rheumatoid Arthritis (RA)

RA is a chronic autoimmune disease characterized by the inflammation of synovial joints and the destruction of cartilage and bone. The presence of anti-citrullinated protein antibodies (ACPAs) is a hallmark of RA, and their generation is heavily influenced by Tfh cells. Elevated numbers of Tfh cells have been identified in the blood and synovial fluid of RA patients, suggesting that they play a central role in sustaining chronic inflammation [44,45,51]. Tfh cells in RA promote ACPA production by stimulating autoreactive B cells in ectopic GCs within synovial tissue, contributing to localized inflammation and tissue damage [52].

In RA, the cytokine milieu further promotes Tfh-cell activity. IL-21 and IL-6 are both elevated in RA patients and act synergistically to enhance Tfh-cell function and sustain B-cell differentiation within the synovial microenvironment. IL-6, in particular, is implicated in the initial activation of Tfh cells, whereas IL-21 sustains their activity, creating an inflammatory feedback loop that amplifies autoimmune responses [51,52,53]. Moreover, Tfh cells in RA express high levels of ICOS, a co-stimulatory molecule that reinforces Tfh–B-cell interactions. Blocking ICOS/ICOS-L signaling in RA has shown potential to reduce Tfh-mediated inflammation, highlighting ICOS as a therapeutic target in autoimmune diseases with Tfh dysregulation [51,52]. Among Tfh subsets, Tfh17 cells appear to have the strongest correlation with disease severity in RA, likely due to their dual role in promoting B-cell activation and driving Th17-mediated inflammation, which is well documented in RA pathology [53].

The presence of ACPAs and the rheumatoid factor (RF) is also a hallmark of RA. Tfh cells help autoreactive B cells, promoting their differentiation into plasma cells that produce these autoantibodies. Increased Tfh activity is directly associated with higher ACPA and RF titers, which contribute to joint inflammation and tissue damage in RA. Other studies [51,52,54] demonstrated that the frequency of Tfh cells in RA patients was positively correlated with ACPA levels, indicating that Tfh cells play a critical role in driving autoantibody-mediated pathology. Given the role of Tfh cells in ACPA production, therapies targeting Tfh–B-cell interactions are a promising area of investigation in RA. Rituximab, a B-cell depleting agent that targets CD20 on B cells, has demonstrated efficacy in rheumatoid arthritis (RA) by disrupting the Tfh–B-cell interaction cycle. By depleting B cells, rituximab indirectly impairs the cooperation between Tfh cells and B cells, which is essential for processes such as B-cell activation, differentiation, and antibody production. However, new therapies are being developed to specifically target Tfh-specific pathways, potentially offering more selective modulation of immune responses without the need for broad B cell depletion. The use of ICOS inhibitors or IL-21 receptor blockers could provide a more nuanced approach to controlling Tfh-cell activity in RA, especially for patients who do not respond to conventional therapies [53,54].

### 6.3. Tfh Cells in Anti-Neutrophil Cytoplasmic Antibodies (ANCA)-Associated Vasculitis (AAV)

AAV encompasses autoimmune conditions that involve small-vessel inflammation, driven by ANCA, which target proteins within neutrophils, leading to their activation and recruitment, release inflammatory mediators, and damage vessel walls [20]. Recent studies have revealed a role for Tfh cells in promoting ANCAproduction and sustaining the autoimmune response in AAV [55].

In patients with active AAV, circulating Tfh cells are significantly increased and correlate with ANCA titers, suggesting that Tfh cells actively drive disease activity. IL-21, predominantly produced by Tfh cells, is elevated in AAV and contributes to the differentiation of B cells into ANCA-producing plasma cells. This cytokine-driven interaction highlights Tfh cells as central players in maintaining the chronic inflammatory state characteristic of AAV [56]. Moreover, ectopic lymphoid structures have been observed in inflamed tissues of AAV patients, providing a local environment for Tfh–B-cell interactions and further promoting ANCA production.

Studies such as [57] support the therapeutic potential of targeting IL-21 in AAV. Blocking IL-21 reduces ANCA levels and diminishes disease severity in preclinical models, suggesting that IL-21 inhibition may alleviate inflammation without compromising other immune functions. Given the link between Tfh cells, IL-21, and ANCA production, an IL-21 blockade could become a targeted strategy for treating AAV, potentially slowing disease progression and reducing relapse rates [55,56].

In addition to IL-21, other Tfh-associated pathways, such as PD-1 and ICOS, are being explored for their potential roles in AAV. For instance, impairments in PD-1 signaling, which typically act as a checkpoint to limit Tfh-cell activity, may contribute to the persistence of autoreactive Tfh cells in AAV. Impairments in PD-1-signaling can be caused by a variety of factors, including genetic mutations, epigenetic modifications, or chronic immune activation. In autoimmune diseases like AAV, persistent antigenic stimulation can lead to the downregulation of PD-1 expression on T cells, or the loss of PD-1 functionality due to alterations in downstream signaling pathways. Additionally, inflammatory cytokines such as IL-6 and TNF-α, which are often elevated in AAV, may also contribute to PD-1 dysfunction. These changes can result in an inability to properly regulate Tfh-cell activity, leading to the persistence of autoreactive Tfh cells, which are a key player in driving the autoimmune response in AAV. Enhancing PD-1 signaling could help restore immune tolerance and suppress ANCA production, providing a novel therapeutic approach for managing AAV [57,58].

The role of Tfh cells in autoimmune diseases like SLE, RA, and AAV underscores their importance as mediators of immune dysregulation and highlights the potential for targeted therapies. Through their interactions with B cells, Tfh cells facilitate the production of high-affinity autoantibodies, perpetuating inflammation and tissue damage. Cytokines like IL-21 and IL-6 play crucial roles in these processes, both promoting Tfh-cell survival and enhancing their pathogenic effects. Therapeutic strategies aimed at disrupting Tfh–B-cell interactions, blocking Tfh-derived cytokines, or modulating co-stimulatory pathways (e.g., ICOS and PD-1) may offer effective means of managing autoimmune diseases driven by Tfh-cell dysregulation. Understanding Tfh cells’ diverse roles in a normal immune response and in various autoimmune settings continues to inform new, precision-targeted treatments with the potential to reduce disease activity and improve patient outcomes.

The recent studies on ANCA-associated vasculitis (AAV) have added significant depth to the understanding of Tfh cells in autoimmune pathology. Older studies [57] highlighted that elevated Tfh cells in Granulomatosis with Polyangiitis (GPA), a form of AAV, are associated with increased ANCA titers and more severe disease manifestations. These findings suggest that Tfh cells could be used as biomarkers for disease activity and potentially to predict flares in AAV patients. By showing that IL-21 produced by Tfh cells drives ANCA production, this study reinforces the potential of IL-21 blockade as a therapeutic approach in AAV, which could help mitigate the chronic vascular inflammation and immune dysregulation characteristic of the disease. Namilumab is an IL-21 blockade which is a monoclonal antibody that targets and inhibits the IL-21 receptor (IL-21R). By blocking IL-21R signaling, namilumab can reduce the activation and proliferation of immune cells such as Tfh cells and B cells, which are implicated in autoimmune diseases like AAV [59].

These insights into the role of IL-21 in supporting ANCA-producing B cells highlight Tfh cells as central players in AAV. Together, these studies suggest that modulating Tfh activity or blocking IL-21 could provide new therapeutic options for AAV, aligning Tfh cells with targeted interventions that reduce disease burden without broader immunosuppressive effects [60,61].

## 7. Therapeutic Implications in Humans

The central role of Tfh cells in promoting autoreactive B-cell responses makes them an attractive target for therapeutic intervention in autoimmune diseases. Several strategies have been proposed to modulate Tfh-cell activity, either by directly targeting Tfh cells or by disrupting their interactions with B cells.

### 7.1. Targeting IL-21 Signaling

Given the critical role of IL-21 in Tfh-cell function, blocking IL-21 signaling is a promising approach for reducing autoantibody production in autoimmune diseases. Clinical trials are currently underway to evaluate the efficacy of IL-21 receptor antagonists in diseases such as SLE and RA [62]. In addition, therapies targeting IL-21 may be beneficial in AAV, where elevated IL-2 levels are associated with increased ANCA production and disease activity [63].

### 7.2. Bcl-6 Inhibitors

As the master regulator of Tfh-cell differentiation, Bcl-6 represents another potential therapeutic target. Bcl-6 inhibitors have been shown to reduce Tfh-cell numbers and suppress GC formation in preclinical models of autoimmune diseases [63,64]. However, targeting Bcl-6 must be approached with caution, as this transcription factor also regulates other aspects of immune function.

### 7.3. PD-1/PD-L1 Modulation

The PD-1/PD-L1 pathway plays a critical role in regulating Tfh-cell activity and preventing excessive GC responses. Enhancing PD-1 signaling in autoimmune diseases could help to suppress the overactivity of Tfh cells and reduce autoantibody production [65,66]. However, the challenge lies in balancing the suppression of autoreactive Tfh cells with the preservation of normal immune responses, as PD-1 signaling is also important for maintaining tolerance to infections and tumors [67].

Most clinical studies, targeting Tfh function and their interaction with B cells, have been performed in patients with SLE, and are demonstrated in Table 2.

## 8. Conclusions

T-follicular helper (Tfh) cells play a central role in the immune system by facilitating the development of high-affinity antibody responses through their interactions with B cells in **GCs**. In the context of autoimmune diseases, however, these cells become key players in immune dysregulation and pathogenic autoantibody production. Their ability to drive B-cell maturation and antibody generation makes them indispensable for effective immunity but also makes them liable to promote the progression of autoimmunity when dysregulated. This dual nature of Tfh cells—crucial for both protective and pathological immune responses—has made them a focal point in the study of diseases such as SLE, RA, and AAV.

A major contribution of Tfh cells to autoimmune diseases is through their production of cytokines, particularly IL-21, which supports the proliferation and differentiation of autoreactive B cells. Elevated levels of circulating Tfh-like cells and IL-21 have been observed in patients with SLE, RA, and AAV, correlating strongly with disease severity and autoantibody levels. The feedback loop between IL-21 and Tfh cells not only sustains Tfh-cell function but also drives the continuous expansion of autoreactive B cells, leading to the production of pathogenic antibodies that damage tissues and drive chronic inflammation. By understanding these interactions, researchers have identified potential therapeutic targets, with the IL-21 blockade emerging as a promising strategy in managing autoimmune disease symptoms and slowing progression.

The role of Tfh cells in the regulation of humoral immunity and their contribution to autoimmune diseases has been increasingly recognized over the past decade. Through their interactions with B cells, Tfh cells promote the production of high-affinity antibodies, a process that is crucial for the clearance of pathogens. However, when dysregulated, Tfh cells drive the production of autoantibodies that target self-antigens, leading to chronic inflammation and tissue damage.

In autoimmune diseases, such as SLE, RA, and AAV, the overactivation of Tfh cells and the subsequent breakdown of tolerance mechanisms lead to the production of pathogenic autoantibodies that drive disease progression. In SLE, elevated levels of circulating Tfh cells and the overproduction of IL-21 contribute to the expansion of autoreactive B cells, resulting in the production of anti-dsDNA and anti-Sm antibodies. Similarly, in RA, Tfh cells play a central role in the generation of ACPAs, which drive synovial inflammation and joint destruction.

The identification of Tfh cells in AAV is particularly noteworthy, as it extends our understanding of the role of Tfh cells beyond traditional autoimmune diseases characterized by systemic inflammation. In AAV, the expansion of circulating Tfh cells and the production of IL-21 are associated with increased ANCA titers and disease activity. These findings suggest that Tfh cells are directly involved in driving the pathogenic B-cell responses that lead to vascular inflammation and damage in AAV.

Looking to the future, Tfh-cell research offers promising opportunities for targeted therapies that modulate Tfh activity without compromising immune function. As our understanding of Tfh-cell regulation expands, therapies that selectively inhibit pathogenic Tfh responses while preserving protective immunity are likely to emerge. The development of subtype-specific inhibitors and cytokine blockers, along with Tfh cell-related biomarkers could transform autoimmune disease treatment, leading to more effective care for patients with chronic autoimmune conditions. However, challenges remain in ensuring that these therapies do not induce excessive immunosuppression, leaving patients vulnerable to infections or blunting responses to vaccines.

## Figures and Tables

**Table 1:** Phenotypic and functional differences between secondary lymphoid Tfh and circulating Tfh cells.

	Secondary Lymphoid Tfh Cells	Circulating Tfh (cTfh) Cells
**(1) Location**	GC of lymph nodes, spleen	Peripheral blood
**(2) Phenotype:**		
**-CXCR5**	High	Moderate to high
**-PD-1**	High	Low
**-Bcl-6**	High	Low
**-ICOS**	High	Low
**-CD45RO**	No	Moderate
**-CXCR3**	Undetermined	
**0**	Undetermined	
**(3) Functions:**	Involved in active immune responses, direct interaction with B cells in GC through CD40L	Memory cell repertoire
	B-cell proliferation, class switch, high-affinity B cells	B cells help upon recall
		Can migrate to GC
	Undetermined role in infection and vaccination	Up-regulation of immune response
**Cytokines produced**	IL-21, IL-4, IL-10	IL-21

**Table 2:** Completed and ongoing clinical studies, designed to evaluate the effect of monoclonal antibodies against certain molecules on Tfh cells [68,69,70,71,72,73,74,75].

Study Code	Drug	Target Molecule	Tfh Cells	Phase	Status	References
**NCT03371251**	avizakimab	IL-21	Inhibit Tfh-cell production	Phase I	active	
**NCT02106897**	BIIB059 (Litifilimab)	Dendritic cell antigen 2 (BDCA2)	Inhibit Tfh-cell differentiation	Phase I	Completed	[68]
**NCT02847598**	BIIB059 (Litifilimab)	Dendritic cell antigen 2 (BDCA2)	Inhibit Tfh-cell differentiation	Phase II	Completed	[68]
**NCT04961567**	BIIB059 (Litifilimab)	Dendritic cell antigen 2 (BDCA2)	Inhibit Tfh-cell differentiation	Phase III	Active	[68]
**NCT06570798**	Inebilizumab	Dnti-CD19	Inhibit Tfh–B-cell reactions	Phase II	Active	[69]
**NCT04976322**	Dapirolizumab Pegol	Targets CD40L	Inhibit Tfh–B-cell reactions	Phase II	Completed	[70]
**NCT05048238**	*Tofacitinib*	JAK Inhibitors	Inhibit Tfh-cell differentiation	Phase I	Completed	[71]
**NCT02535689**	*Ttofacitinib*	JAK Inhibitors	Inhibit Tfh-cell differentiation	Phase I	Completed	[71]
**NCT03312335**	Aldesleukin	Low-dose hrIL-2	Supress Bcl-6	Phase II	Completed	
**NCT04835441**	Acazicolcept	ICOS	Inhibit Tfh–B-cell reactions	Phase II	Active	[72]
**NCT04058028**	AMG 570	ICOS	Inhibit Tfh–B-cell reactions	Phase II	Completed	[73]
**NCT04184258**	Mesenchymal stem cells		Regulate Tfh-cell function	Phase I/II	Completed	[74,75]
